# Induction of immunoglobulin transcription factor 2 and resistance to MEK inhibitor in melanoma cells

**DOI:** 10.18632/oncotarget.17866

**Published:** 2017-05-15

**Authors:** Eun-Hye Hur, Bon-Kwan Goo, Juhyun Moon, Yunsuk Choi, Jung Jin Hwang, Choung-Soo Kim, Kyun Seop Bae, Jene Choi, Suk Young Cho, Sang-Hwa Yang, Jeongbeob Seo, Gilnam Lee, Je-Hwan Lee

**Affiliations:** ^1^ Department of Hematology, Asan Medical Center, University of Ulsan College of Medicine, Seoul, Korea; ^2^ Division of Hematology and Hematological Malignancies, Ulsan University Hospital, University of Ulsan College of Medicine, Ulsan, Korea; ^3^ Institute for Innovative Cancer Research, Asan Medical Center, University of Ulsan College of Medicine, Seoul, Korea; ^4^ Department of Urology, Asan Medical Center, University of Ulsan College of Medicine, Seoul, Korea; ^5^ Department of Clinical Pharmacology and Therapeutics, Asan Medical Center, University of Ulsan College of Medicine, Seoul, Korea; ^6^ Department of Pathology, Asan Medical Center, University of Ulsan College of Medicine, Seoul, Korea; ^7^ Wuxi App Tec (Shanghai) Co., Ltd. Shanghai, China; ^8^ Department of Biotechnology, College of Life Science and Biotechnology, National Creative Research Initiatives Center for Inflammatory Response Modulation, Yonsei University, Seoul, Korea; ^9^ Department of Medicinal Chemistry, CHABIOMED Co., LTD., Seongnam-Si, Korea; ^10^ MD Healthcare, Inc., Seoul, Korea

**Keywords:** MAPK, resistance, ITF-2, beta-catenin, melanoma

## Abstract

Primary or acquired resistance to MEK inhibitors has been a barrier to successful treatment with MEK inhibitors in many tumors. In this study, we analyzed genome-wide gene expression profiling data from 6 sensitive and 6 resistant cell lines to identify candidate genes whose expression changes are associated with responses to a MEK inhibitor, selumetinib (AZD6244). Of 62 identified differentially expressed genes, we selected *Immunoglobulin Transcription Factor 2*, also known as transcription factor 4 as a potential drug resistance marker for further analysis. This was because the ITF-2 expression increase in resistant cell lines was relatively high and a previous study has suggested that *ITF-2* functions as an oncogene in human colon cancers. We also established an AZD6244 resistant cell line (M14/AZD-3) from an AZD6244 sensitive M14 cell line. The expression of the ITF-2 was elevated both in primary AZD6244 resistant cell line, LOX-IMVI and acquired resistant cell line, M14/AZD-3. Targeted silencing of *ITF-2* by siRNA significantly enhanced susceptibility to AZD6244 in resistant cells. Wnt/β-catenin pathway was activated through direct interaction of p-ERK and GSK3β. Our results suggest that up-regulation of the *ITF-2* gene expression is associated with cellular resistance to MEK inhibitors, and activation of Wnt signaling pathway through interaction of p-ERK and GSK3β seems to be a mechanism for increase of ITF-2.

## INTRODUCTION

Intracellular growth-promoting signals are transduced through the activation of the small G protein RAS, which activates RAF and then activates mitogen-activated protein kinase (MAPK) kinase (MEK), leading to the phosphorylation and activation of MAPK (originally called extracellular signal-regulated kinase [ERK]). MEK1 and MEK2 show a unique specificity for ERK1/2 and the ERKs have wide spectrum substrates, many of which play a role in promoting cell proliferation and survival [1]. Small-molecule inhibitors of one or more components of the RAS-MAPK signaling pathway have potential therapeutic properties for some cancers, particularly those that have a dysregulated MAPK pathway (for example, *B-RAF*-mutated melanoma and *K-RAS*/*B-RAF*-mutated colorectal cancer) [2]. MEK inhibitors inhibit MEK1 and/or MEK2, homologous dual specificity kinases that share ERK as their only known catalytic substrate [3]. PD098059 was the first MEK inhibitor to be described [4] and several other MEK inhibitors have since been developed in clinical use for various cancers [5]. The Food and Drug Administration (FDA) of the United States recently approved one of these agents, trametinib for the treatment of patients with unrespectable or metastatic melanomas harboring *B-RAF*^V600E^ or *B-RAF*^V600K^ mutations, as detected by an FDA-approved test (https://www.fda.gov/downloads/drugs/guidancecomplianceregulatoryinformation/guidances/ucm481951.pdf). AZD6244 (Selumetinib) is a non-ATP competitive inhibitor and is highly specific for MEK1/2. Multiple phase 2 studies of this drug have been conducted in patients with a wide range of cancers [5].

Although recent studies have demonstrated impressive antitumor activities of MEK inhibitors, many tumors show intrinsic resistance of these agents. Reliable biomarkers of susceptibility or resistance to MEK inhibitors are thus needed to allow tailoring of individualized treatments with these compounds and to reduce the risk of unnecessary drug toxicities. It has been suggested that activating mutations in the *B-RAF* gene represent the most important predictive biomarker for sensitivity to MEK inhibitors [6, 7]. However, early clinical data have revealed that *B-RAF* mutant tumors were not uniformly responsive to MEK inhibitors although tumors with these mutations seemed to be more sensitive. [8, 9] Furthermore, the duration of the responses to MEK inhibitors is reported to be relatively short (median ≤ 5 months) in melanoma [10]. Other biomarkers such as *RAS* mutations or ERK activation correlate poorly with the sensitivity of cells to MEK inhibitors [11, 12].

The NCI-60 is a set of 60 human cancer cell lines derived from diverse tissues, including brain, blood and bone marrow, breast, colon, kidney, lung, ovary, prostate, and skin. These cell lines have now been subjected to a battery of experiments including extensive pharmacological characterization analyses via treatment with over 100,000 chemical compounds, chromosome karyotyping, and gene expression analysis using various DNA microarray platforms [13]. The current approaches to studying the genetic basis of cancer are exploring substantial components of the expressed genome rather than focusing on individual genes [14]. Researchers can now use standards-based repositories such as the Stanford Microarray Database and the Gene Expression Omnibus, which have developed to store and disseminate the results of microarray experiments [15].

The proto-oncogene β-catenin has been linked to the pathogenesis of hepatocellular carcinoma, colorectal carcinoma, lung cancer, malignant breast tumors, and leukemia through the Wnt-signal transduction pathway [16] *Immunoglobulin Transcription Factor-2 (ITF-2)* is one of several specific target genes of T-cell factor-dependent transcription upon translocation of β-catenin to the nucleus [17].

In our current study we aimed to screen for novel response predictive markers for the AZD6244, MEK inhibitor by analyzing published microarray data from AZD6244 sensitivity profiling of the NCI-60 cell lines and also aimed to validate the candidate markers in both primary and acquired resistance models.

## RESULTS

### Screening of cancer cell lines for their response to a MEK inhibitor, AZD6244

We performed assays for the growth response of a panel of NCI-60 cell lines to 10 μM or less AZD6244 by proliferation assay. On the basis of the response to AZD6244, we selected 6 sensitive (IC_50_ ≤ 0.5 μM) and 6 resistant (IC_50_ > 5 μM) cell lines (Supplementary Figure 1). The IC_50_ values for the 6 resistant cell lines ranged from 6.032 to 125.9 μM, while the 6 sensitive cell lines showed IC_50_ values of less than 0.5 μM (range, 0.02923 to 0.4870 μM). One resistant and 5 sensitive cell lines harbored a *B-RAF* mutation; and 1 resistant and 1 sensitive line contained a *K-RAS* mutation. One resistant cell line had an *N-RAS* mutation (Table 1).

**Table 1:** Characteristics of 12 cell lines: 6 cell lines were sensitive to a MEK inhibitor, AZD6244, and 6 cell lines were resistant to the agent

	Cell line	Origin	Gene mutation			IC_50_ for AZD6244 (μM)
			*B-RAF*	*K-RAS*	*N-RAS*	
Resistant	SF-295	Glioblastoma	-	-	-	7.383
	SNB-19	Glioblastoma	-	-	-	15.13
	LOX-IMVI	Melanoma	V600E	-	-	6.032
	K-562	CML	-	-	-	82.07
	CCRF-CEM	ALL	-	G12D	wt	125.9
	HL-60	APL	-	-	Q61L	20.95
Sensitive	UACC-62	Melanoma	V600E	-	-	0.4870
	UACC-257	Melanoma	V600E	-	-	0.4650
	M14	Melanoma	V600E	-	-	0.06103
	SK-MEL-28	Melanoma	V600E	-	-	0.2098
	COLO-205	Colon cancer	V600E	-	-	0.02923
	SW-620	Colon cancer	-	G12V	-	0.05538

CML, chronic myeloid leukemia; ALL, acute lymphoblastic leukemia; APL, acute promyelocytic leukemia.

### Selection of *ITF-2* as a potential AZD6244 resistance marker from public microarray data

Following public microarray data acquisition and analysis, a total of 62 differentially expressed genes (DEGs) were selected from 12 cell lines (6 AZD6244 resistant; CCRF-CEM, HL-60, K-562, LOX-IMVI, SF-295 and SNB-19 vs. 6 AZD6244 sensitive; COLO-205, SW-620, M14, SK-MEL-28, UACC-257 and UACC-62) (Supplementary Figure 2). Of these selected genes, 18 were up-regulated and 44 were down-regulated in AZD6244 resistant cell lines (Supplementary Table 1). Table 2 lists the top 10 AZD6244 resistant signature genes and we confirmed the expression using quantitative real-time PCR for these 10 genes (data not shown). We selected the *Immunoglobulin transcription factor-2* (*ITF-2*) gene because it showed the strongest upregulation in resistant lines and previous studies have suggested that this gene is a downstream target of the Wnt/β-catenin/T-cell factor pathway [18].

**Table 2:** Top 10 resistant signature genes for AZD6244

Symbol	Description	Entrez ID	Fold-change*
*ITF-2*	Immunoglobulin transcription factor 2	6925	5.91
*ZNF22*	zinc finger protein 22	7570	5.66
*TGFB1*	transforming growth factor, beta 1	7040	5.62
*ZEB1*	zinc finger E-box binding homeobox 1	6935	5.58
*ASF1A*	ASF1 anti-silencing function 1 homolog A	25842	3.58
*BCAT1*	branched chain amino-acid transaminase 1, cytosolic	586	3.57
*RANBP6*	RAN binding protein 6	26953	2.78
*SACS*	spastic ataxia of Charlevoix-Saguenay	26278	2.63
*MAP3K4*	mitogen-activated protein kinase kinasekinase 4	4216	2.45
*NAA15*	N(alpha)-acetyltransferase 15, NatA auxiliary subunit	80155	2.39

*Fold-change of mean expression intensities in resistant group/sensitive group.

### Increased expression of ITF-2 in cell lines having primary or acquired resistance to AZD6244

**Figure 1:**
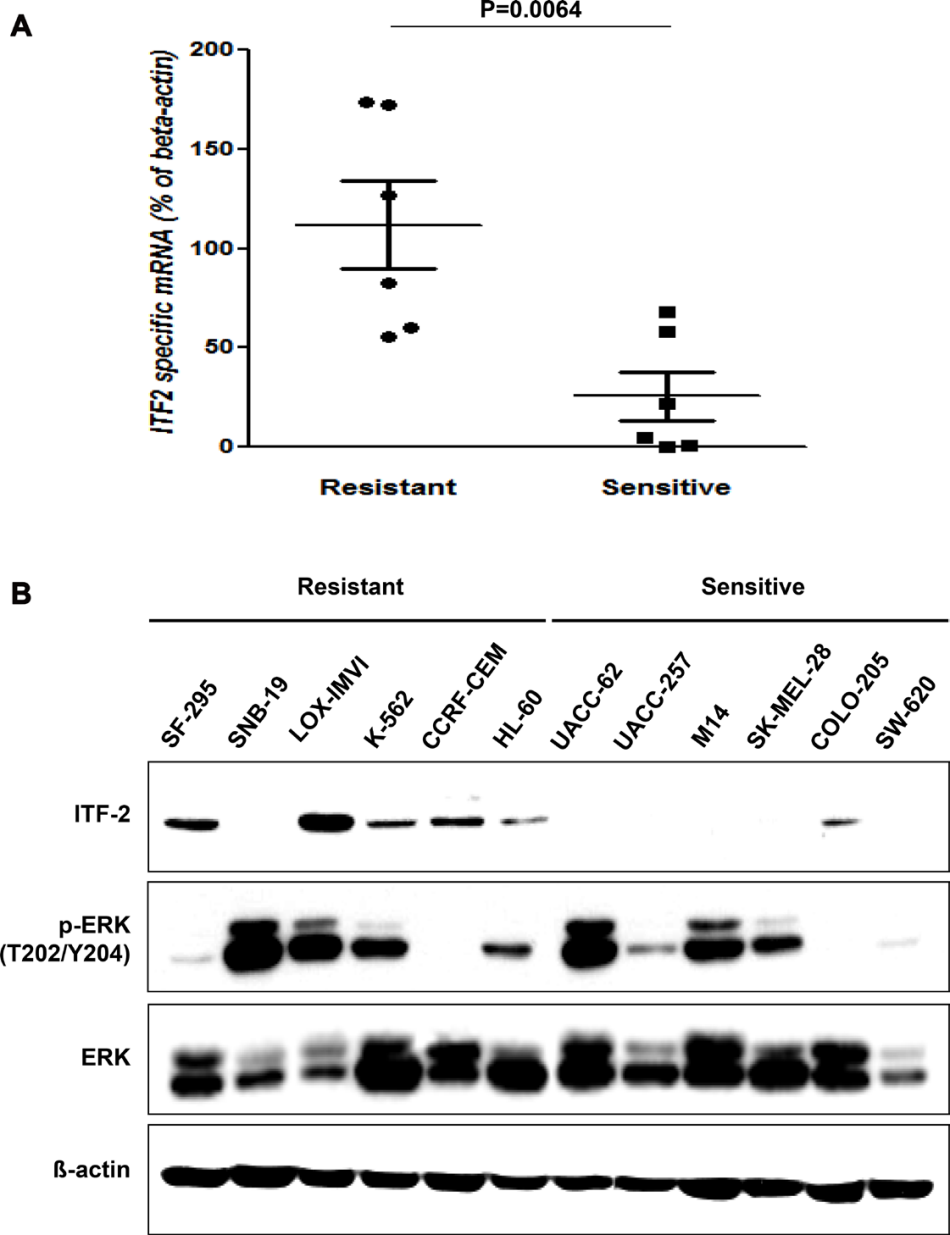
Basal levels of ITF-2 in AZD6244 resistant and sensitive cell lines (**A**) Relative expression of endogenous *ITF-2* mRNA in each indicated cell line based on the expression in SW620. Each sample was tested in triplicate, and gene expression levels were normalized to those of β-actin mRNA. The mRNA levels of *ITF-2* in AZD6244 resistant cell lines were significantly higher than those in sensitive cell lines. Statistical significances are calculated by the Mann-Whitney *U*-test. (**B**) Equal amounts of total cellular proteins were subjected to western blot analysis for ITF-2, and phospho-specific and total ERK1/2. ITF-2 was elevated in AZD6244 resistant cell lines except SNB-19, but the p-ERK levels were not significantly different according to the sensitivity to AZD6244. Beta-actin was included as a loading control.

The mRNA levels of *ITF-2* in AZD6244 resistant cancer cell lines were found to be significantly higher than in cell lines sensitive to this inhibitor (Figure 1A). Moreover, western blot using an anti-ITF-2 monoclonal antibody (mAb) showed that the ITF-2 protein level was elevated in 5 of 6 AZD6244 resistant cell lines but in only one sensitive cell line. The p-ERK levels were not significantly associated with the sensitivity to AZD6244 (Figure 1B).

**Figure 2:**
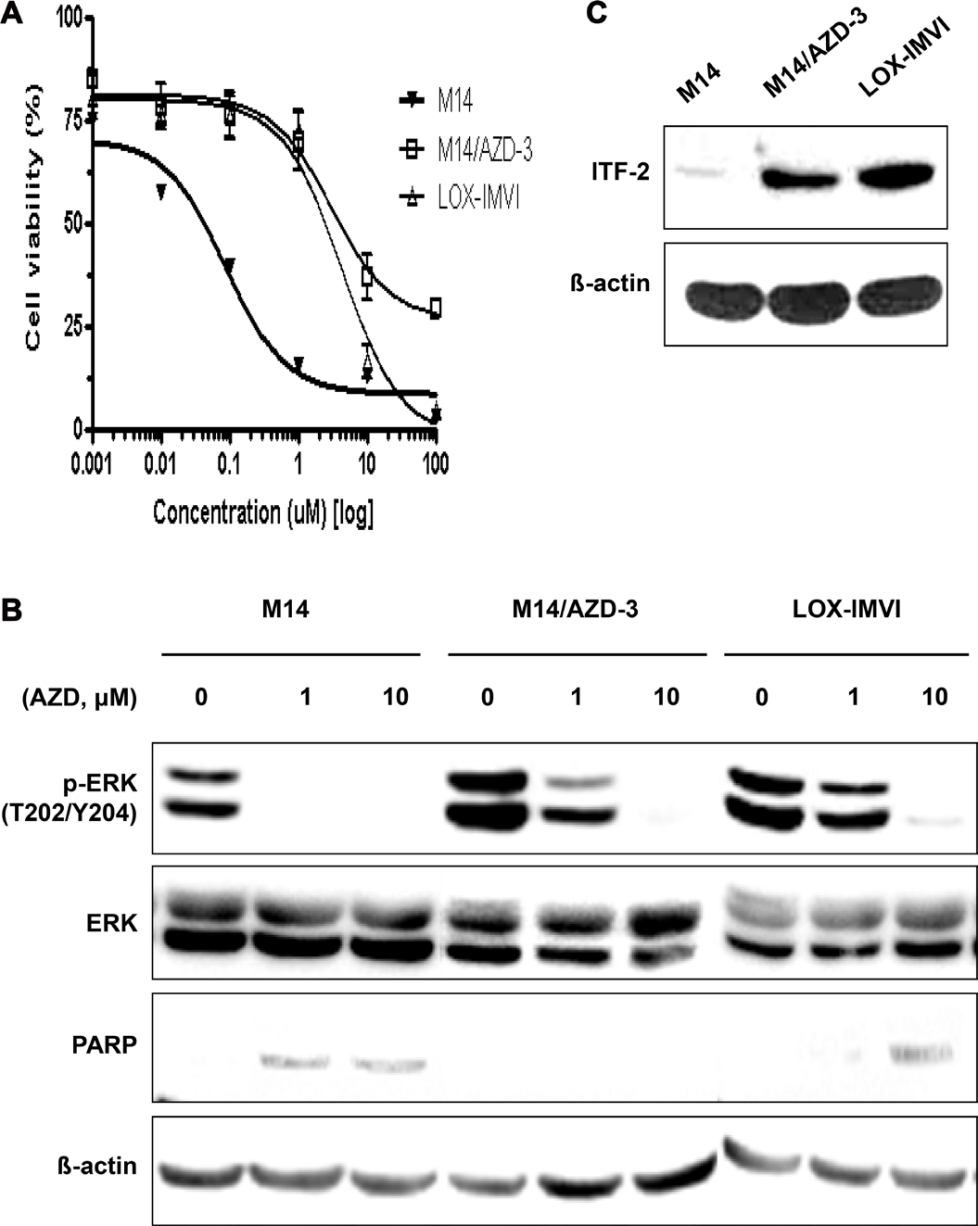
Establishment of an acquired AZD6244 resistant cell line, M14/AZD-3 and overexpression of ITF-2 in M14/AZD-3 (**A**) M14/AZD-3 cell line was established by long-term treatment of an AZD6244 sensitive melanoma cell line, M14, with increasing dose of AZD6244. (**B**) With 1 μM of AZD6244, suppression of phosphorylated ERK (p-ERK) and cleavage of poly (ADP-ribose) polymerase (PARP) did occur in M14, but not in M13/AZD-3 (acquired AZD6244 resistant cell line) and LOX-IMVI (primary AZD6244 resistant cell line). (**C**) Overexpression of ITF-2 protein in M14/AZD-3 and LOX-IMVI.

M14/AZD-3 is 33.4-fold more resistant to AZD6244 compared to parent M14 (Figure 2A). Direct sequencing demonstrated that both M14 and M14/AZD-3 had *B-RAF*^V600E^ mutation, but did not have mutations in *MEK1* exon 3 and exon 6 which encompass the validated resistant alleles (Supplementary Figure 3) [51]. M14/AZD-3 needed higher concentration of AZD6244 to decrease the p-ERK level and did not induce poly (ADP-ribose) polymerase (PARP) cleavages following treatment of AZD6244 (Figure 2B). Western blot demonstrated that the ITF-2 protein level was elevated in M14/AZD-3 as well as LOX-IMVI, while it was not in parent M14 cell line (Figure 2C). ITF-2 protein overexpression in both primary (LOX-IMVI) and acquired (M14/AZD-3) resistant cell lines suggested the possible role of ITF-2 in resistance to AZD6244. In contrast, the levels of RAS/RAF/MEK/ERK signaling proteins were not significantly changed in resistant cell lines (M14/AZD-1, M14/AZD-3, and M14/AZD-4) compared to M14 (Supplementary Figure 4).

### Suppression of *ITF-2* by siRNA in primary and acquired resistant cell lines

**Figure 3:**
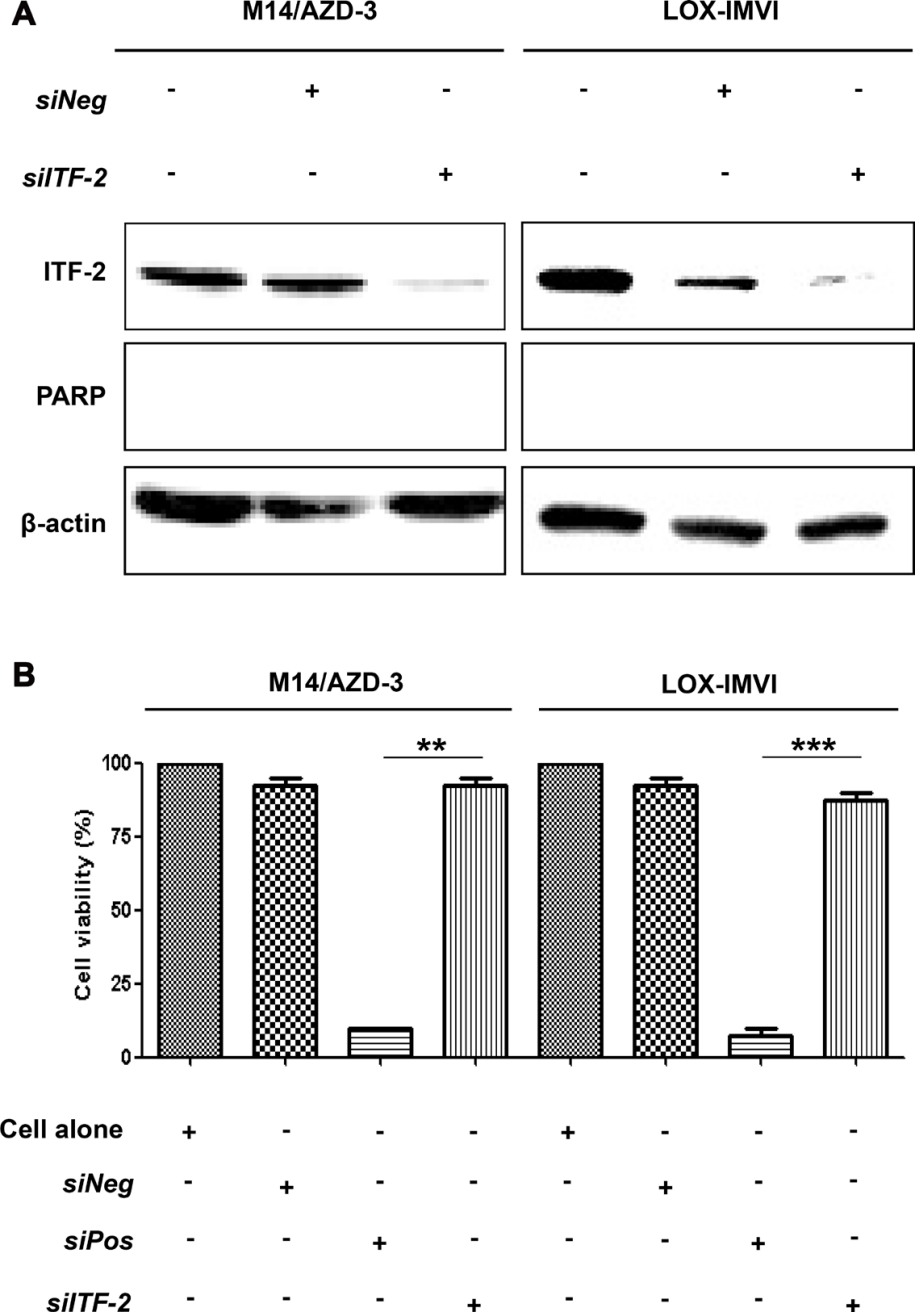
Suppression of *ITF-2* by siRNA in primary and acquired AZD6244 resistant cell lines M14/AZD-3 and LOX-IMVI cells were transfected with *ITF-2* siRNA (*siITF-2*), non-targeting control siRNA (*siNeg*) and cell death control siRNA (*siPos*). All siRNAs were used as 50 nM concentrations for 24 h. (**A**) Endogenous *ITF-2* mRNA levels decreased by the transfection of *siITF-2*, but transfection alone did not induce cleavage of poly (ADP-ribose) polymerase (PARP). (**B**) The transfection of *siITF-2* did not influence on the cell viability in resistant cell lines. **P = 0.001, ***P < 0.001.

To exam whether ITF-2 overexpression is associated with the resistance to AZD6244, M14/AZD-3 and LOX-IMVI cells were transfected with either 50 nM of control siRNAs or siRNA against *ITF-2* (*siITF-2*) (Figure 3). Transfection of *siITF-2* into M14/AZD-3 and LOX-IMVI effectively reduced ITF-2 protein expression, but transfection alone did not induce cleavage of PARP (Figure 3A) or did not inhibit cell proliferation (Figure 3B).

**Figure 4:**
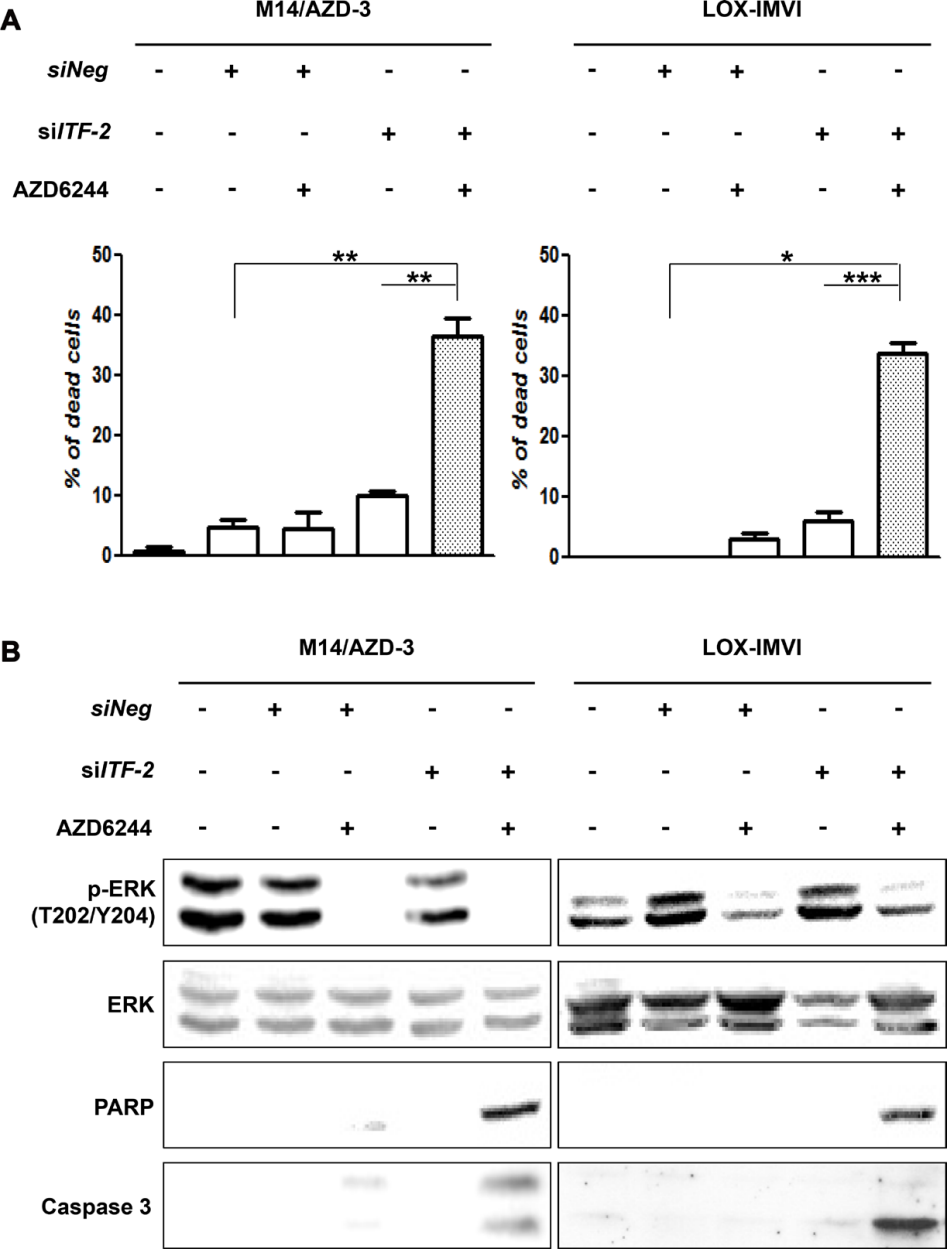
Combination of AZD6244 and siRNA against *ITF-2* The cytotoxic and apoptotic effects were assessed after treatment of AZD6244 resistant cells with 10 μM of AZD6244 for 24 h following transfection of siRNA against *ITF-2* (*siITF-2*). (**A**) Live-Dead cell staining showed dramatically increased proportion of dead cells by combination of AZD6244 and *siITF-2*. *P = 0.002, **P = 0.001, ***P < 0.001. (**B**) Western blot analysis also demonstrated increased cleavage of procaspase-3 and poly (ADP-ribose) polymerase (PARP) with treatment of AZD6244 following transfection of *siITF-2* (Figure 4B).

Although the transfection of *siITF-2* alone did not induce the death of AZD6244 resistant cells, it might alter response of the resistant cells to AZD6244. To test this hypothesis, we treated AZD6244 resistant cells with 10 μM of AZD6244 for 24 h following transfection of *siITF-2*. The Live-Dead cell staining showed that ITF-2 suppression by transfection of *siITF-2* dramatically increased the proportion of dead cells with treatment of AZD6244 (Figure 4A). Western blot analysis also demonstrated increased cleavage of procaspase-3 and PARP with treatment of AZD6244 following transfection of *siITF-2* (Figure 4B).

This phenomenon could also be found in other primary AZD6244 resistant cell line, human glioma-derived SF-295. The cell line had increased *ITF-2* level, which was effectively suppressed by two different siRNAs against *ITF-2* (*siITF-2* #1 and *siITF-2* #2) (Supplementary Figure 5A). The apoptotic and cytotoxic effects of AZD6244 on SF-295 cells were enhanced by transfection of both siRNAs as evidenced by inhibition of phosphorylated ERK (p-ERK) and increased cleavage of PARP in western blot (Supplementary Figure 5B) and increase of the proportion of dead cells in Live-Dead cell staining (Supplementary Figure 5C).

These results suggest that the ITF-2 suppression could enhance the susceptibility to AZD6244 in melanoma cells with primary or acquired resistance.

### Activation of Wnt signaling pathway in primary and acquired resistant cell lines

**Figure 5:**
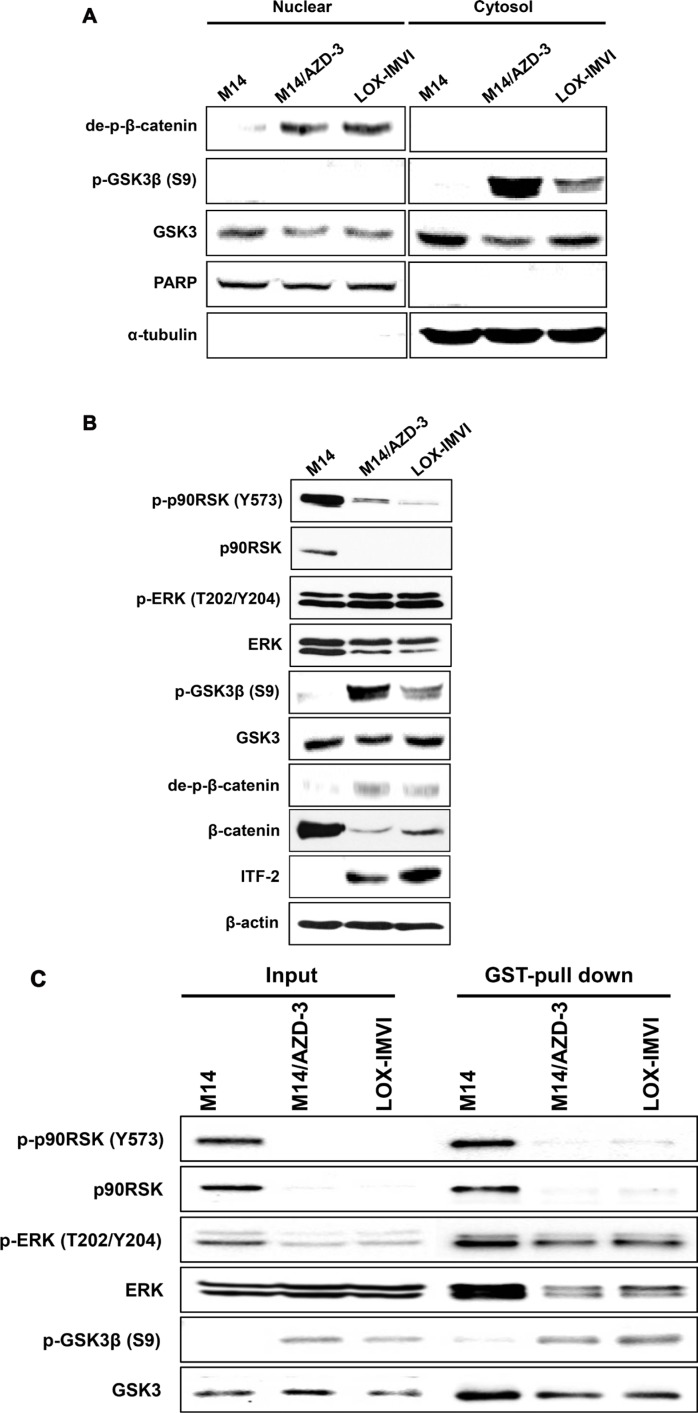
ITF-2 induction by activation of Wnt signaling pathway (**A**) Western blot analysis for Wnt/β-catenin signaling proteins demonstrated that dephosphorylated β-catenin was translocated to the nucleus and phosphorylated Ser9 of GSK3β was accumulated in cytosol fraction in AZD6244 resistant cell lines (M14/AZD-3 and LOX-IMVI). (**B**) Western blot analysis showed that the p90RSK was downregulated in AZD6244 resistant cell lines. (**C**) GST pull-down assay using pGEX-GSK3β and Glutathione-Sepharose 4B (GST) beads demonstrated the direct interaction of p-ERK and GSK3β and phosphorylation of GSK3β at Ser9 in AZD6244 resistant cell lines (M14/AZD-3 and LOX-IMVI).

Because ITF-2 is known as a downstream target of Wnt signaling pathway, we examined the pathway in AZD6244 sensitive cell line (M14) and resistant cell lines (M14/AZD-3 and LOX-IMVI). Translocation of dephosphorylated β-catenin to the nucleus and accumulation of phosphorylated Ser9 of GSK3β in cytosol fraction were found in AZD6244 resistant cell lines (M14/AZD-3 and LOX-IMVI), but not in AZD6244 sensitive cell line, M14 (Figure 5A). The same findings were observed in SF-295 (Supplementary Figure 6). These findings suggest that activation of Wnt signaling pathway due to inactivation of GSK3β leads to increased transcription of ITF-2 in AZD6244 resistant cells. Because GSK3β is known to be phosphorylated at Ser9 by the most downstream kinase of the classical MAPK cascade, called ribosome S6 kinase (RSK), [19] we assayed total and phosphorylated p90RSK (Tyr573). Interestingly, we found that p90RSK was downregulated in AZD6244 resistant cell lines (Figure 5B).

We assumed that p-ERK might directly interact with GSK3β and we performed GST pull-down assay using pGEX-GSK3β and Glutathione-Sepharose 4B (GST) beads. GSK3β fused GST bead was interacted with phosphorylated p90RSK (Tyr573) and ERK (T202/Y204) in M14 but the interaction did not activate phosphorylation of GSK3β (S9). In contrast, the bead was interacted with phosphorylated ERK (T202/Y204) and phosphorylation of GSK3β (S9) was induced in both resistant cell lines without p90RSK (Figure 5C). The findings demonstrated that p-ERK was directly bound to GSK3β, which was phosphorylated at Ser9, in AZD6244 resistant cell lines (M14/AZD-3 and LOX-IMVI).

## DISCUSSION

Drug resistance, either primary or acquired, is one of the most challenging aspects of anticancer agents. A critical component of cancer drug development is the identification of biomarkers that can be used to predict resistance to these agents. Biomarker identification is also important for minimizing patient exposure to potentially toxic agents for little clinical benefit and to develop rational approaches for overcoming drug resistance. Many studies have been under taken to gain an improved understanding of the mechanisms of cancer drug resistance and have identified factors that seemed to be associated with resistance to MEK inhibitors, [20] including *MEK1* mutations, [21] over-expression of microphthalmia-associated transcription factor (MITF), [22] aberrant expression of musculoaponeurotic fibrosarcoma (MAF), [23] concurrent activation of the phosphoinositide 3-kinase (PI3K)/AKT/mammalian target of rapamycin (mTOR) pathway [24, 25] or the signal transducer and activator of transcription 3 (STAT3) pathway, [26] and an ‘invasive phenotype’ of transcriptional pathway signatures [27]. In our current study, we obtained evidence that the over-expression of *ITF-2* might be a novel biomarker of cellular resistance to MEK inhibitors because the mRNA and protein levels of this gene in AZD6244 resistant cell lines were significantly elevated and suppression of *ITF-2* by siRNA enhanced the susceptibility of AZD6244 resistant cells to this inhibitor. These results were observed with both primary AZD6244 resistance cell line (LOX-IMVI) and acquired resistance cell line (M14/AZD), which was established as a model for acquired resistance using a sensitive cell line harboring *B-RAF* mutation. Our study results might provide a clue to prevent or overcome the development of acquired resistance to MEK inhibitors, which is a common and serious problem in real clinical settings.

**Figure 6:**
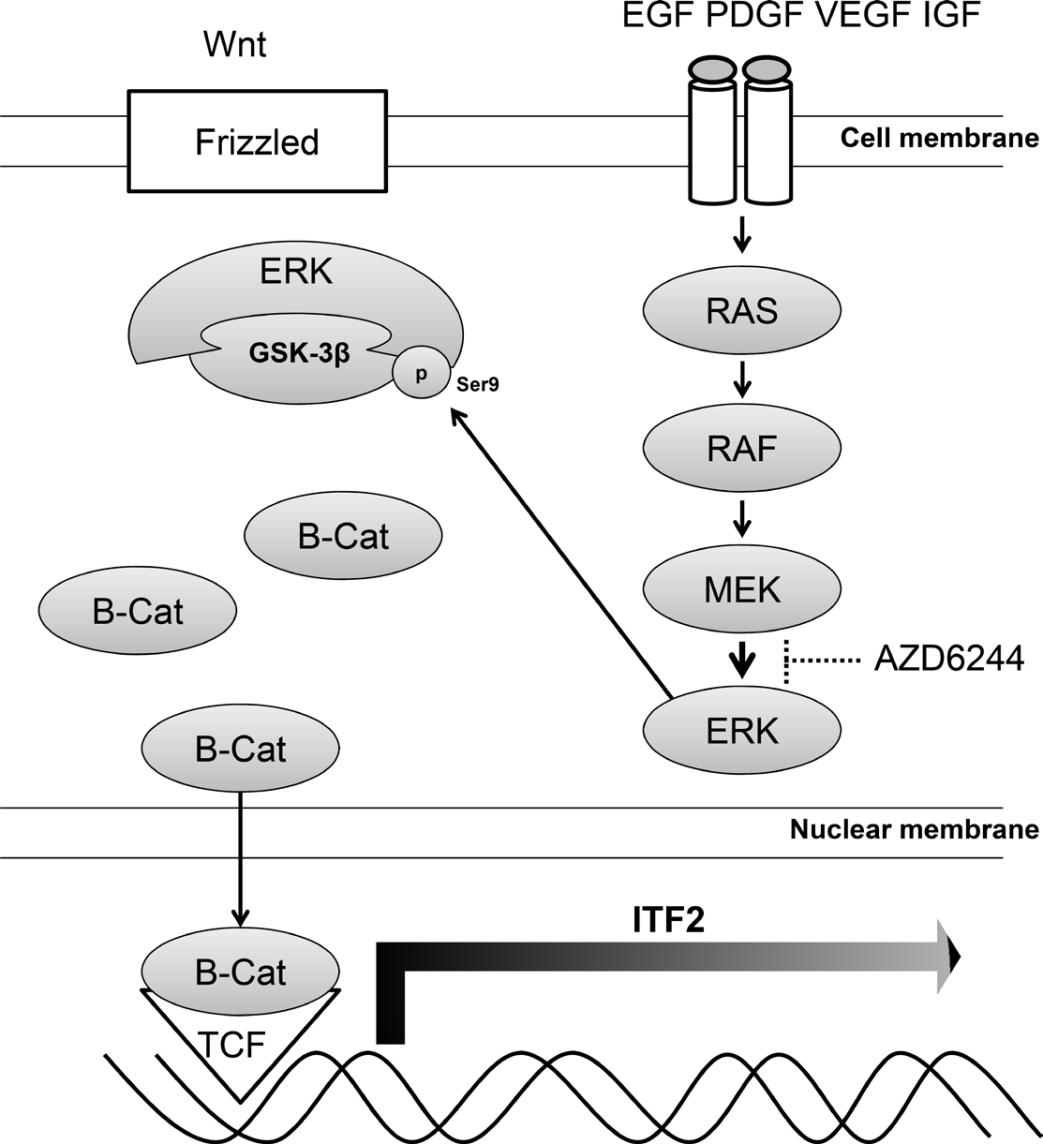
Model of the ITF-2 induction via ERK/GSK3β/β-catenin In AZD6244 resistant cells, p-ERK directly interacts with GSK3β, leading to phosphorylation at Ser9 and dephosphorylation of β-catenin. The dephosphorylated β-catenin is translocated to the nucleus and the association of β-catenin and T-cell factor results in the transcription of *ITF-2* gene, one of Wnt target genes.

The *ITF-2* gene (also known as *TCF4*, *E2-2*, *ME2*, or *SEF2*) is located on human chromosome 18q21, consists of 20 exons (exons 1 and 20 are noncoding), and spans 360 kb. *ITF-2* belongs to the class A subfamily of basic helix-loop-helix (bHLH) transcriptional regulators [28, 29]. This gene is different from *T-cell transcription factor 4* on human chromosome 10q25.3, which was previously termed *TCF4*, but is now designated as *TCF7L2*. The *ITF-2* gene encodes two splice variants, *ITF-2A* and *ITF-2B*. *ITF-2A* is a truncated splice variant with a low transcriptional potency. *ITF-2B* produces a larger protein, which is ubiquitously expressed and dimerizes with other classes of bHLH proteins to regulate tissue-specific gene expression through E-box sites in various cell types including myocytes, osteoblasts, B and T lymphocytes, and neuronal cells [30]. Previous studies have demonstrated that ITF-2 is a downstream target of the Wnt/β-catenin/ *TCF7L2* pathway, which is frequently disrupted in human cancers [17, 18, 31]. It is also well known that nuclear transcriptional activity of β-catenin is enhanced upon sustained oncogenic stimulation by the RAS-MAPK signaling pathway [32]. Like *c-MYC* and *cyclin D1*, *ITF-2* functions as an oncogene when deregulated in human colon cancer or ovarian cancer, [18, 31] although other studies have reported that the inactivation of *ITF-2* may play a role in early gastric carcinoma progression or in the colonic adenoma to carcinoma transition [33, 34]. In another earlier study aiming to identify predictive biomarkers of the response to AZD6244 in colorectal cancer, members of the Wnt signaling pathway were found to be highly over-expressed in response to AZD6244 and shRNA knockdown experiments indicated the functional involvement of these factors in mediating resistance to this inhibitor [35]. In our study, we observed increase of inactive form of GSK3β and translocation of β-catenin to nucleus in AZD6244 resistant cell lines (M14/AZD3 and LOX-IMVI), leading to increased transcription of ITF-2. It has been known that GSK3β is phosphorylated at Ser9 by stress-activated p90RSK [36]. In our study, however, p90RSK was downregulated in AZD6244 resistant cell lines and GST pull-down assay showed that p-ERK was bound to GSK3β, which was phosphorylated at Ser9. Thus, it is reasonably assumed that p-ERK directly interacts with GSK3β leading to phosphorylation at Ser9, and activation of Wnt pathway results in the transcription of *ITF-2* gene in AZD6244 resistant cells (Figure 6). Downstream signals of ITF-2 related to resistance to MEK inhibitor remain to be studied.

MEK inhibitors have generally shown limited effectiveness as monotherapeutic agents and combination regimens are a possible treatment approach to overcome MEK inhibitor resistance. Indeed, simultaneously inhibition of the MAPK and PI3K/AKT/mTOR pathways has been proposed for melanoma [37]. Moreover, in a *K-RAS* mutant lung cancer mouse model, a combination of a MEK inhibitor at doses below the maximum tolerated dose with a PI3K/AKT/mTOR inhibitor was found to suppress the growth of the tumors [38]. In another previous *in vitro* study using melanoma cell lines, a high degree of cross-resistance to a B-RAF inhibitor (vemurafenib) and a MEK inhibitor (AZD6244) was reported, and genetic or pharmacological inhibition of the PI3K/AKT/mTOR pathway was found to partially or completely reverse this resistance [39]. Furthermore, a combination of AZD6244 and an BCL2 inhibitor (ABT-263) was shown to be synergistic in causing a cell death response in colon cancer or melanoma cells harboring *B-RAF* or *RAS* mutations, and also to delay the onset of acquired resistance and in some cases overcome acquired resistance to AZD6244 [40]. The MAPK pathway is activated by *BCR-ABL* in nilotinib-resistant chronic myeloid leukemia (CML) cells and nilotinib acts synergistically with MEK inhibitors to kill drug-resistant CML cells both *in vitro* and *in vivo* [41]. The available evidence suggests that cytotoxic chemotherapeutic agents can activate the MAPK pathway through diverse processes and that MEK inhibitors may effectively blocked the activation [42]. It has also been demonstrated that there are synergistic or additive effects of MEK inhibitors in combination with various cytotoxic chemotherapeutic agents. *In vitro* studies using leukemia cell lines have reported that MEK inhibitors enhance the cytotoxic effects of cytarabine or doxorubicin [43, 44]. Similar enhanced effects of MEK inhibitors in combination with cytotoxic drugs have been observed in xenograft tumor models of solid cancers including melanoma, [45] and biliary, [46] colon, [47, 48] lung, [48] pancreatic [48] or hepatocellular carcinoma [49]. When MEK inhibitors are combined with traditional chemotherapeutic agents, drug scheduling may have a critical disease management role because MEK inhibitors cause cell cycle arrest whereas many standard cytotoxic agents kill actively dividing cancer cells [46, 48]. In our current study, a combination of AZD6244 and *ITF-2* siRNA induced cell death in AZD6244 resistant cell lines, although the magnitude of enhanced susceptibility to AZD6244 was not adequate for clinical application, probably due to the only partial suppression of *ITF-2* expression by siRNA. Wnt/ β-catenin pathway inhibitors might suppress ITF-2 more effectively and may provide a novel combination approach for MEK inhibitors.

In summary, we have identified the *ITF-2* gene as a novel resistance biomarker for an MEK inhibitor, AZD6244. The mRNA and protein levels of ITF-2 were found to be significantly elevated in cell lines with primary or acquired resistance to AZD6244 and a targeted silencing of *ITF-2* by siRNA significantly enhanced the susceptibility of primary resistant cells (LOX-IMVI) and acquired resistant cells (M14/AZD-3) to AZD6244. Activation of Wnt signaling pathway through direct interaction of p-ERK and GSK3β seems to be a mechanism for increase of ITF-2.

## MATERIALS AND METHODS

### Cancer cell lines

The NCI-60 cell lines were purchased from the Jackson Laboratory (Bar Harbor, ME) and were cultured at 37°C in 5% CO_2_ in RPMI-1640 medium containing 5% or 10% (v/v) fetal bovine serum and 1% penicillin/streptomycin (Invitrogen, Carlsbad, CA). Information regarding the mutation status of the *B-RAF*, *K-RAS*, and *N-RAS* genes of each cell line was obtained from the Wellcome Trust Sanger institute website (http://cancer.sanger.ac.uk/cosmic/sample/overview?id=1295340, http://cancer.sanger.ac.uk/cosmic/sample/overview?id=1238117).

### Establishment of an AZD6244 resistant melanoma cell line

Human melanoma M14 cell line was sensitive to AZD6244 *in vitro*. M14 cells were continuously exposed to gradually increasing concentration of AZD6244 up to 500 nM. An AZD6244 resistant subclone was isolated using limiting dilution method and AZD6244 resistant cell lines (M14/AZD-1, M14/AZD-3, and M14/AZD-4) could be established. In most experiments, M14/AZD-3 was used.

### Cell proliferation assay

Cell viability was assessed by the luminescent-based CellTiter-Glo system (Promega, Madison, WI) and Cell counting kit -SK (Dojindo Molecular Technologies, Inc. Rockville, MD) in accordance with the manufacturer's instructions. Briefly, cells were plated at 1,000–3,000 per well in a 96-well opaque plate and were incubated in complete growth medium. Cells were treated with various concentrations of MEK1/2 inhibitor (AZD6244), which was synthesized by Chemizon (Seongnam, Korea) and prepared as a 50 mmol/L stock solution in DMSO. After 48 h, cell viability was determined by measuring luminescent signals with a VICTOR^™^ X Light luminescence plate reader (PerkinElmer, Waltham, MA). The concentration of AZD6244 required for 50% growth inhibition was scored as the half maximal inhibitory concentration (IC_50_) values.

### Public microarray data acquisition and data analysis

To identify candidate genes associated with resistant or sensitive responses to AZD6244 genome-wide gene expression profiling data from Affymetrix HG-U133A chips were downloaded from the Gene Expression Omnibus (www.ncbi.nlm.nih.gov/geo). The chip data were analyzed with RNA-eXpress (www.rnaexpress.org) using its default analysis settings and a quintile normalization method. Analyses of preprocessed data were performed using BRB-ArrayTools (linus.nci.nih.gov/BRB-ArrayTools.html). A two-sample *t*-test was performed between sensitive and resistant groups at *P* < 0.005 to select for DEGs between the two groups.

### Genomic DNA PCR and direct sequencing

Genomic DNA was purified using the QIAamp DNA mini kit (Qiagen, Valencia, CA). All PCR amplifications were performed with HotStar HiFidelity Polymerase kit (Qiagen, Valencia, CA) following the manufacturer's instructions. *B-RAF* codon V600 was PCR amplified using following primers: 5′GGCCAAAAATTTAATCAGTGGA3′ and 5′ AGCCTCAATTCTTACCATCC3′. 50 μl PCR reactions were 35 cycles at 95°C for 30 sec, 56°C for 30 sec, 72°C 45 sec. The *MEK1* genetic mutation was accomplished using published methods [51]. The sequencing primers were the same as the PCR forward primers and the sequencing reaction was performed using standard-seq single (Macrogen Inc. Seoul. Kr).

### Quantitative real-time RT-PCR

Total RNAs were extracted using TRIzol^®^ Reagent (Invitrogen) and converted into cDNA using a QuantiTect Reverse Transcription Kit (Qiagen, Valencia, CA). The transcript levels were quantified via the SYBR Green real time PCR method using LightCycler^®^ 480 SYBR Green I Master Mix (Roche Applied Science) on a Roche-480 system using primer sets (Supplementary Table 2). After an initial denaturation step at 95°C for 5 min, amplification occurred over 45 cycles of denaturation at 95°C for 30 s, annealing at 60°C for 30 s, and extension at 72°C for 30 s. Each sample was tested in triplicate, and gene expression levels were normalized to those of *beta-actin*.

### Inhibition of gene expression by siRNA transfection

The siRNA was used for targeted silencing of *ITF-2*. Because off-target activity of siRNA may complicate the interpretation of phenotypic effects in gene silencing, two kinds of *ITF-2* siRNA's were used for the experiments: *siITF-2 #1* (Integrated DNA technologies, Inc., Coralville, IA) and *siITF-2* #2 (Santa Cruz Biotechnology, Inc., Dallas, TX). The level of off-target effects was assessed by western blot analysis using an anti-ITF-2 mAb in M14/AZD-3 and both *ITF-2* siRNA's significantly reduced ITF-2 expression levels (Supplementary Figure 7). A non-targeting control siRNA (negative control) and a cell death control siRNA (positive control) were purchased from Qiagen. Cells were seeded at 2 × 10^5^ per well in a 6-well plate and were transfected with 50 μM of siRNA using jetPRIME^®^ in accordance with the manufacturer's instructions (Polyplus, Illkirch, France). Cells were then incubated at 37°C for 24 h.

### Western blot analysis

Cells were lysed with lysis buffer (cell signaling technology, Beverly, MA) and protein samples (50 μg) were separated by SDS-PAGE and were blotted onto polyvinylidene difluoride membranes (Millipore, Bedford, MA). After blocking with 5% (w/v) non-fat dry milk powder for 1 h, the membranes were incubated with primary antibodies overnight at 4°C, then with secondary antibody conjugated with horseradish peroxidase (Enzo Life Sciences, Farmingdale, NY). Specific antigen-antibody complexes were detected by enhanced chemiluminescence using SuperSignal West Pico Chemiluminescent Substrate (Thermo Fisher Scientific, Waltham, MA). Specific antibodies were as follows: ITF-2 (Abcam, Cambridge, MA), phospho-beta-catenin (p-beta-catenin), beta-catenin, phospho-ERK (p-ERK), ERK, phospho-GSK3 beta (Y216 and S9), GSK-3 alpha and beta, phospho-90RSK (Y573), 90RSK, PARP, cleaved caspase-3 (Cell Signaling Technology, Beverly, MA), dephospho-beta-catenin (de-p-beta-catenin) (Enzo bioscience Inc. France), KRAS, B-RAF, phosphor-MEK, MEK, and beta-actin (Sigma-Aldrich, St. Louis, MO).

To isolate cytoplasmic and nuclear fractions, cells were incubated using NE-PER^®^ Nuclear and cytoplasmic extraction reagents (Pierce Biotechnology, Rockford, IL) according to the manufacturer's protocol. For comparison, one equivalent of cytoplasmic proteins and two equivalents of nuclear proteins were analyzed side-by-side by western blot analysis with different antibodies.

### Immunofluorescence assay

Cells (2 × 10^5^) grown on sterile glass coverslips were transfected with either non-targeting control siRNA or *ITF-2* siRNA. These transfected cells were then incubated with 1:50 anti-ITF-2 (Santa Cruz Biothchnology, Dallas, TX) at room temperature for 1 h after fixation with 4% paraformaldehyde and permeabilization with triton X-100. The cells were then incubated with secondary anti-goat antibody conjugated with fluorescein isothiocyanate (Bethyl, Montgomery, TX) and counterstained with a 4 μg/mL DAPI solution. After the coverslips were mounted with fluorescence mounting medium (Dako, Carpinteria, CA), cells were viewed using a fluorescence microscope (Olympus-IX71 Olympus, Tokyo, Japan).

### Flow cytometric apoptosis analysis

Cells (1 × 10^6^) were treated with AZD6244 for 24 h, washed twice with cold PBS, and resuspended in 1× Binding Buffer at a concentration of 1 × 10^6^ cells/mL. A 100 μL aliquot of this solution (1 × 10^5^ cells) was then transferred to a 5 mL culture tube to which 5 μL of FITC Annexin V was added. These cells were incubated for 15 min at room temperature in the dark before assaying with an Annexin V–FITC Apoptosis Detection Kit I (BD Biosciences, San Jose, CA), in accordance with the manufacturer's protocol. The percentage of apoptotic cells was analyzed by flow cytometry within 1 h (Becton Dickinson, San Jose, CA).

### Assessment of cell death by Live-Dead cell staining

Cell death after siRNA transfection with or without AZD6244 treatment was assessed using Live-Dead Cell Staining Kit (Enzo Life Sciences). This kit utilizes a cell permeable green fluorescent dye to stain live cells. Dead cells were stained by propidium iodide, a red fluorescent dye, which is actively pumped out of the cytoplasm in viable cells. Stained cells were visualized by fluorescence microscopy (Olympus, Tokyo, Japan).

### GST pull-down assay

pGEX-GSK3β (Addgene, Cambridge, MA) was introduced into *Escherichia coli* BL21(DE3) cells (Stratagene, La Jolla, CA) by transformation and the cells were cultured in 5 ml LB medium at 37°C to the mid-log phase. Protein expression was induced by 1mM isopropyl-1-thio-beta-_D_-galactopyranoside (IPTG). After culturing for 3 h, cells were pelleted by centrifugation and then suspended in 100 μL of a lysis buffer (Cell Signaling Technology, Beverly, MA). Recombinant GST-GSK3β was purified using Glutathione- Sepharose 4B beads (GE Healthcare Life Sciences. UK). Protein lysates of M14, M14/AZD-3, and LOX-IMVI cell lines were respectively combined with 50 μL pre-cleaned Glutathione- Sepharose 4B beads and the mixture was incubated under shaking for 16 h at 4°C. The incubated beads were washed three times with lysis buffer (Cell Signaling Technology, Beverly, MA). After washing, 2× SDS-PAGE loading buffer was added to the washed beads and proteins were extracted from the beads by boiled for 5 min. Proteins were analyzed by 10% SDS-PAGE and western blot analysis.

### Statistical analysis

Statistical differences in cell viability, presented as mean ± standard error of the mean (SEM), were evaluated by the Student *t* test, while those in relative expression of endogenous *ITF-2* mRNA were evaluated by the Mann-Whitney U test. Death rates of two cell lines in assessment of Live-Dead cell staining were compared by a Fisher's exact test. Statistical analyses were performed using SPSS version 21.0 software. For all analyses, the *P* values were 2-tailed, and *P* < 0.05 was considered as statistically significant.

## SUPPLEMENTARY MATERIALS FIGURES AND TABLES




