# Supplementary material for: Induction of immunoglobulin transcription factor 2 and resistance to MEK inhibitor in melanoma cells
Source: Oncotarget. 2017 May 15;8(25):41387–400. doi: 10.18632/oncotarget.17866 (PMC5522248; doi:10.18632/oncotarget.17866)
Supplement: Supplementary file 1 [file oncotarget-08-41387-s001.pdf]

Induction of immunoglobulin transcription factor 2 and resistance to MEK inhibitor in melanoma cells

Supplementary Materials

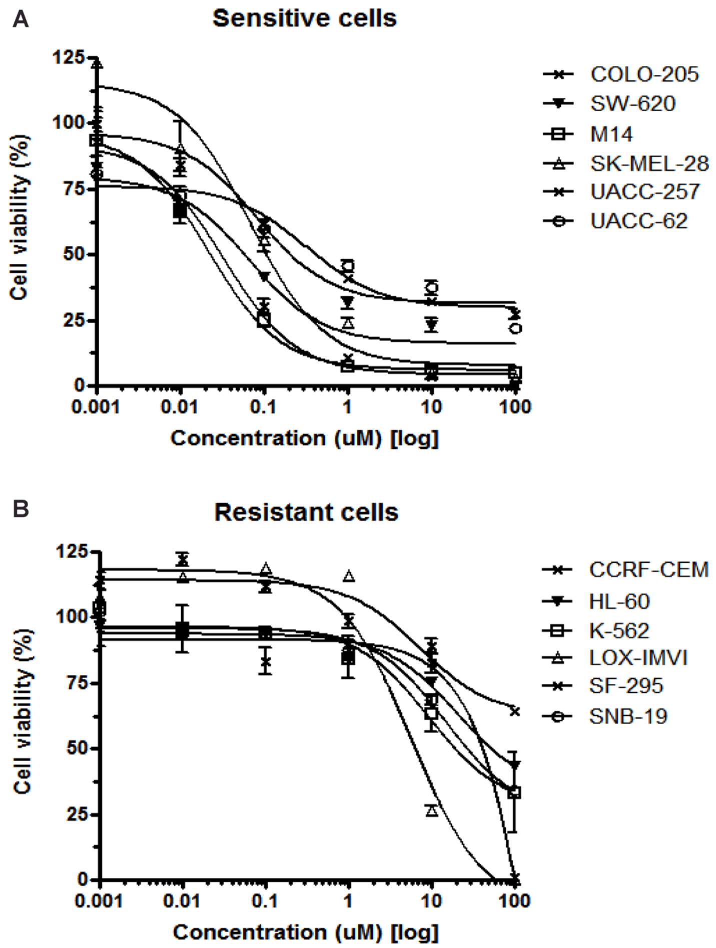

Supplementary Figure 1: Effects of AZD6244 on proliferation of 6 sensitive cell lines and 6 resistant cell lines. *In vitro* cell viability was determined by the luminescent-based CellTiter-Glo system. All cell lines were treated with increasing doses of AZD6244 for 72 hr. Error bars represent the mean ± standard error of mean of at least three independent assays performed.

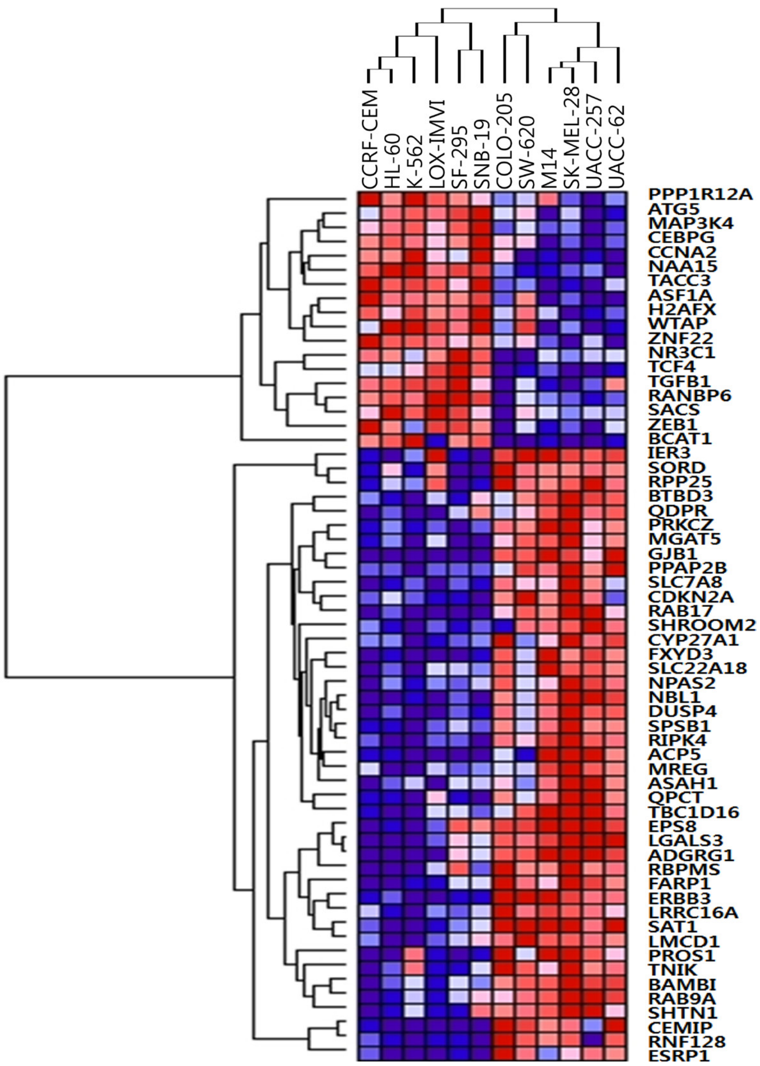

Supplementary Figure 2: A treeview representation of the selected genes and cell lines in this study. Non-treated cell lines and 62 selected genes were subjected to an average-linkage hierarchical clustering based on gene expression levels. The red and blue colors represent up-regulation and down-regulation, respectively, and the relationship between the degree of color change and expression ratio variations are shown in the scale bar.

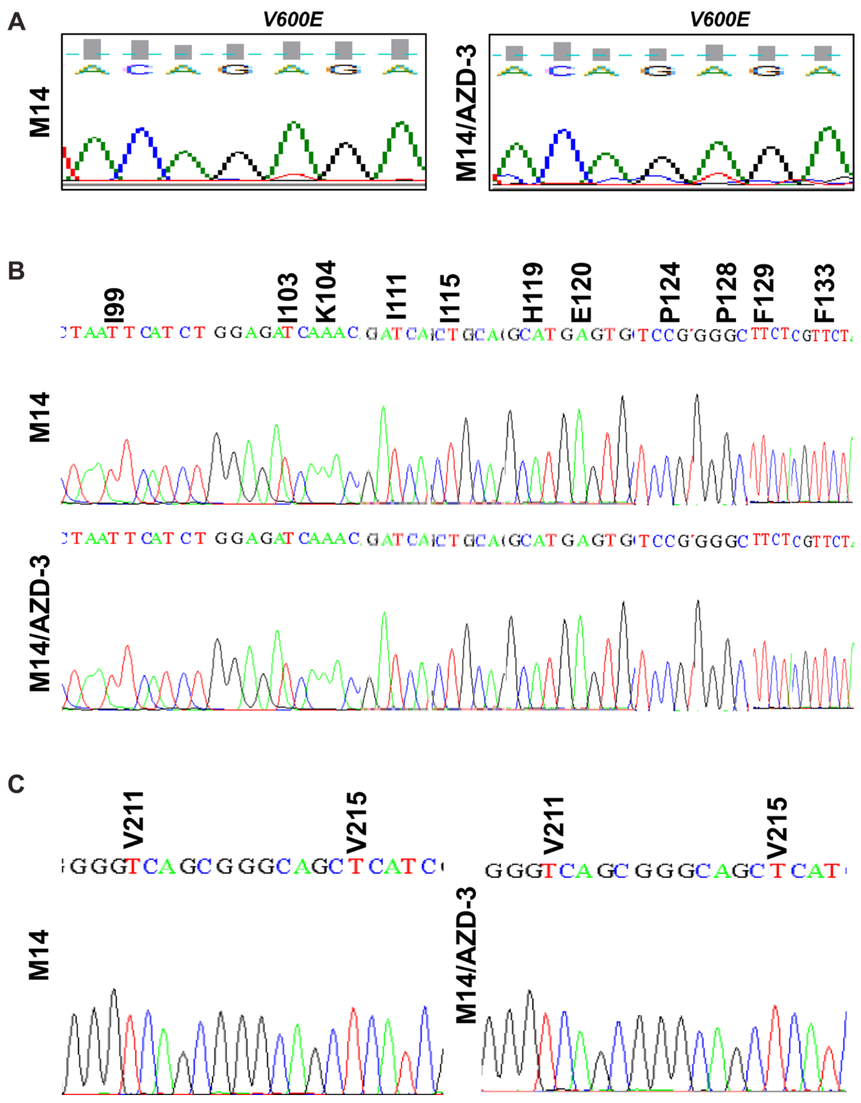

Supplementary Figure 3: *B-RAF* ^{V600E} and *MEK1* gene mutation analysis. The mutation analysis was performed by Sanger sequencing (Macrogen. South Korea) using gDNA. [50, 51] (**A**) *B-RAF* ^{V600E} mutation (**B**) *MEK1* exon 3 coding sequence (**C**) MEK1 exon 6 coding sequence.

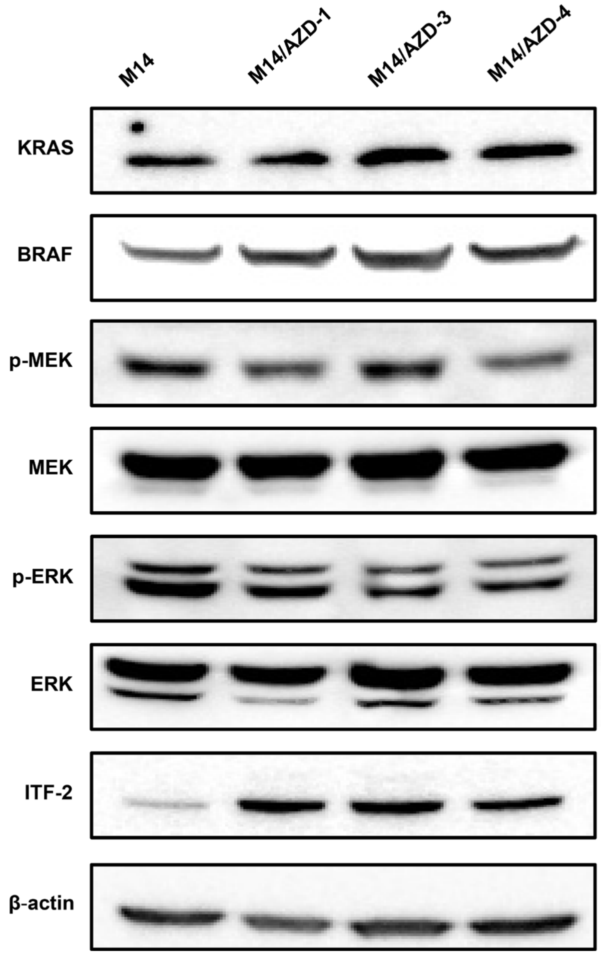

Supplementary Figure 4: RAS signaling molecules and ITF-2in M14 and AZD6244 resistant cell lines. Protein levels of RAS/RAF/MEK/ERK were detected by western blot analysis.

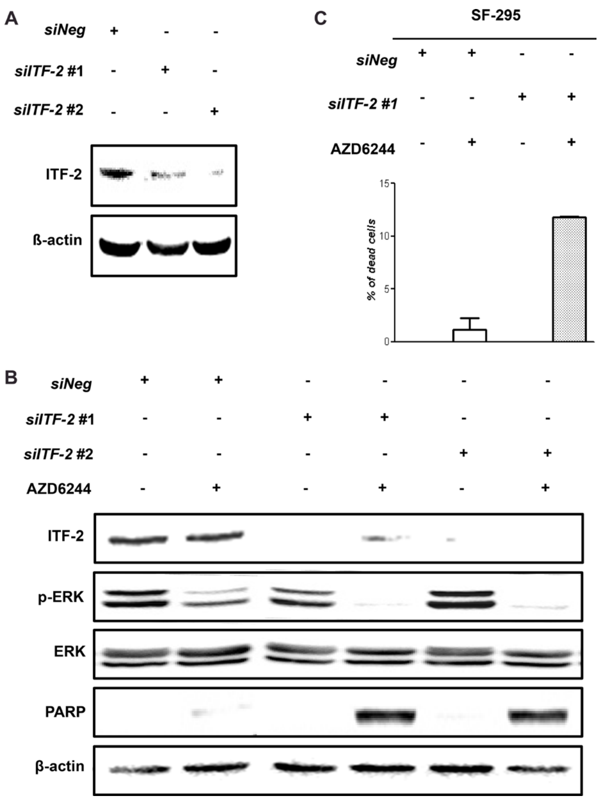

Supplementary Figure 5: Suppression of ITF-2 by two different siRNAs against ITF-2 in primary AZD6244 resistant cell line, human glioma-derived SF-295. (**A**) Both siRNAs (*siITF-2 #1* and *siITF-2* #2) effectively suppressed ITF-2 level. (**B**) Western blot analysis showed inhibition of phosphorylated-ERK (p-ERK) and increased cleavage of poly (ADP-ribose) polymerase (PARP) by treatment of AZD6244 following transfection of *siITF-2* #1 or *siITF-2* #2. (**C**) Live-Dead cell staining showed increased proportion of dead cells with combination of AZD6244 and *siITF-2*.

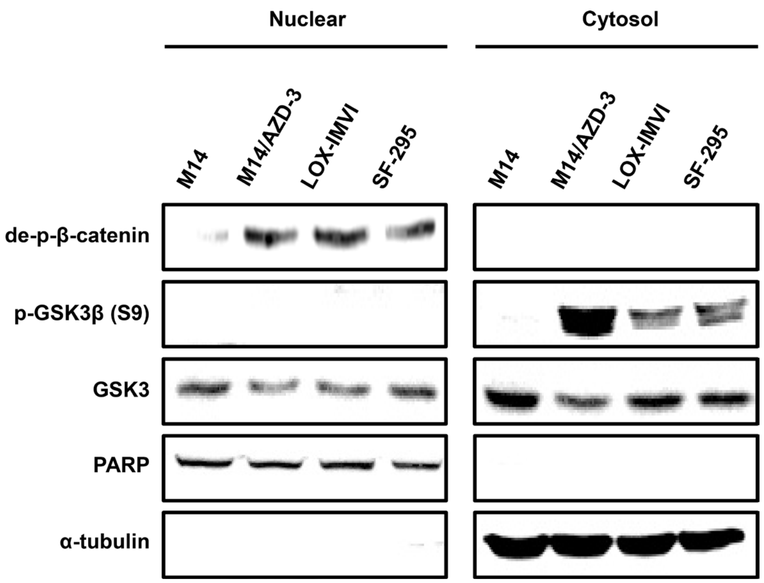

Supplementary Figure 6: Wnt/β-catenin pathway in SF-295. Translocation of dephosphorylated β-catenin to the nucleus and accumulation of phosphorylated Ser9 of GSK3β in cytosol fraction were found in SF-295.

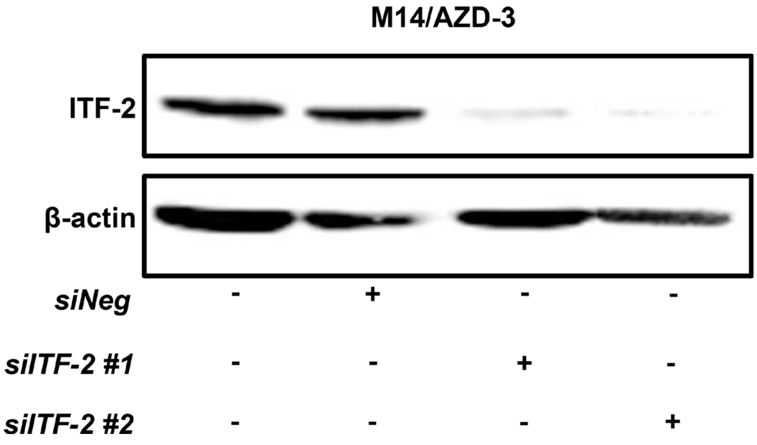

Supplementary Figure 7: siRNA validation. Two siRNA's were used to assess the off-target effects on *ITF-2*.

Supplementary Table 1: Sixty-two genes differentially expressed between AZD6244 sensitive and resistant groups obtained from public microarray data (P < 0.005). See_Supplementary_Table 1

Supplementary Table 2: Primer sequence

Gene	NM#	Forward	Reverse
ITF-2	NM_003199	TGTGTAACACTGAAGCTATGCATTTG	TGCAGCACAACCGAAAACA
TGFB1	NM_000660	CTACCGCTGCTGTGGCTACT	TTCCGCTTCACCAGCTCCAT
ZNF22	NM_006963	ATACATCCCCATGCCCTTGA	AAAGGCTGGCTCAGTTTGTCA
ZEB1	NM_001128128	AGCTTTGTATCTCCTTTGGCC	GCGGGTTAGAGCCTTGAAAA
ASF1A	NM_014034	GAGGAGGAGGGTCAGAACTCG	TACACGGGAGCACTCCACAG
BCAT1	NM_005504	AATCCGCTAGGTCGCGAGTC	CGGCTGCAGCAAGACCTG
RANBP6	NM_012416	AGAGCACTGCAGTCCAACTTCAT	TCATCTCCACCAAACTGTGCCA
SACS	NM_014363	TTCAGCTCACAGCTGCCTCT	CACACTTCCGCAGCACCTG
MAP3K4	NM_005922	GGGATTCAGCACTCGGAGCA	GCTGATGGATTTGGGCAGCA
NAA15	NM_057175	GCGGCAGCGTTAAGTGAGAA	TCCGTAGCTGCTTCAGCCTT
β-actin	NM_001101	CCATCGTCCACCGCAAA	TCAAGAAAGGGTGTAACGCAACTA
