# Supplementary material for: Induction of immunoglobulin transcription factor 2 and resistance to MEK inhibitor in melanoma cells
Source: Oncotarget. 2017 May 15;8(25):41387–400. doi: 10.18632/oncotarget.17866 (PMC5522248; doi:10.18632/oncotarget.17866)
Supplement: Supplementary file 2 [file oncotarget-08-41387-s002.docx]

**Supplementary Table 1.** Sixty-two genes differentially expressed between AZD6244 sensitive and resistant groups obtained from public microarray data (*P*<0.005)

| Entrez ID | Resistant^1^ | Sensitive^2^ | FC^3^ | Symbol | Description |
| --- | --- | --- | --- | --- | --- |
| 6925 | 113.14 | 19.14 | 5.91 | ITF2 | immunoglobulin transcription factor 2 |
| 7570 | 338.37 | 59.74 | 5.66 | ZNF22 | zinc finger protein 22 |
| 7040 | 290.28 | 51.64 | 5.62 | TGFB1 | transforming growth factor, beta 1 |
| 6935 | 164.3 | 29.42 | 5.58 | ZEB1 | zinc finger E-box binding homeobox 1 |
| 25842 | 158.81 | 44.35 | 3.58 | ASF1A | anti-silencing function 1A histone chaperone |
| 586 | 38.63 | 10.84 | 3.57 | BCAT1 | branched chain amino-acid transaminase 1, cytosolic |
| 26953 | 107.89 | 38.87 | 2.78 | RANBP6 | RAN binding protein 6 |
| 26278 | 111.45 | 42.42 | 2.63 | SACS | sacsin molecular chaperone |
| 4216 | 289.45 | 118.11 | 2.45 | MAP3K4 | mitogen-activated protein kinase kinase kinase 4 |
| 80155 | 182.18 | 76.35 | 2.39 | NAA15 | N(alpha)-acetyltransferase 15, NatA auxiliary subunit |
| 1054 | 169.93 | 74.05 | 2.29 | CEBPG | CCAAT/enhancer binding protein (C/EBP), gamma |
| 9589 | 245.74 | 107.08 | 2.29 | WTAP | Wilms tumor 1 associated protein |
| 890 | 398.92 | 181.09 | 2.2 | CCNA2 | cyclin A2 |
| 2908 | 147.89 | 67.32 | 2.2 | NR3C1 | nuclear receptor subfamily 3, group C, member 1 (glucocorticoid receptor) |
| 3014 | 1152.26 | 526.3 | 2.19 | H2AFX | H2A histone family, member X |
| 9474 | 243.74 | 115.45 | 2.11 | ATG5 | autophagy related 5 |
| 10460 | 380.62 | 181.76 | 2.09 | TACC3 | transforming, acidic coiled-coil containing protein 3 |
| 4659 | 122.15 | 60.53 | 2.02 | PPP1R12A | protein phosphatase 1, regulatory subunit 12A |
| 1593 | 160.47 | 324.86 | 0.49 | CYP27A1 | cytochrome P450, family 27, subfamily A, polypeptide 1 |
| 29995 | 59.16 | 121.89 | 0.49 | LMCD1 | LIM and cysteine-rich domains 1 |
| 85002 | 100.95 | 208.91 | 0.48 | *FAM86B1* | family with sequence similarity 86, member B1 |
| 6652 | 270.01 | 562.91 | 0.48 | SORD | sorbitol dehydrogenase |
| 23428 | 176.23 | 363.99 | 0.48 | SLC7A8 | solute carrier family 7 (amino acid transporter light chain, L system), member 8 |
| 80176 | 35.06 | 72.57 | 0.48 | SPSB1 | splA/ryanodine receptor domain and SOCS box containing 1 |
| 4862 | 19.14 | 40.99 | 0.47 | NPAS2 | neuronal PAS domain protein 2 |
| 11030 | 54.09 | 115.09 | 0.47 | RBPMS | RNA binding protein with multiple splicing |
| 5860 | 320.63 | 708.22 | 0.45 | QDPR | quinoid dihydropteridine reductase |
| 9367 | 136.39 | 323.02 | 0.42 | RAB9A | RAB9A, member RAS oncogene family |
| 23043 | 82.5 | 197.84 | 0.42 | TNIK | TRAF2 and NCK interacting kinase |
| 357 | 50.12 | 121.18 | 0.41 | SHROOM2 | shroom family member 2 |
| 55604 | 55.39 | 134.11 | 0.41 | LRRC16A | leucine rich repeat containing 16A |
| 64284 | 59.35 | 144.97 | 0.41 | RAB17 | RAB17, member RAS oncogene family |
| 5590 | 66.28 | 175.32 | 0.38 | PRKCZ | protein kinase C, zeta |
| 1029 | 26.02 | 84.72 | 0.31 | CDKN2A | cyclin-dependent kinase inhibitor 2A |
| 2705 | 84.05 | 276.58 | 0.3 | GJB1 | gap junction protein, beta 1, 32kDa |
| 4249 | 158.06 | 523.16 | 0.3 | MGAT5 | mannosyl (alpha-1,6-)-glycoprotein beta-1,6-N-acetyl-glucosaminyltransferase |
| 54845 | 48.86 | 165.21 | 0.3 | ESRP1 | epithelial splicing regulatory protein 1 |
| 5002 | 105.46 | 368.68 | 0.29 | SLC22A18 | solute carrier family 22, member 18 |
| 22903 | 81.68 | 293.52 | 0.28 | BTBD3 | BTB (POZ) domain containing 3 |
| 54 | 66.12 | 244.85 | 0.27 | ACP5 | acid phosphatase 5, tartrate resistant |
| 10160 | 43.88 | 160.98 | 0.27 | FARP1 | FERM, RhoGEF (ARHGEF) and pleckstrin domain protein 1 (chondrocyte-derived) |
| 54101 | 124.25 | 455.41 | 0.27 | RIPK4 | receptor-interacting serine-threonine kinase 4 |
| 54913 | 80.56 | 299.44 | 0.27 | RPP25 | ribonuclease P/MRP 25kDa subunit |
| 5349 | 78.5 | 344.04 | 0.23 | FXYD3 | FXYD domain containing ion transport regulator 3 |
| 57698 | 39.19 | 168.86 | 0.23 | SHTN1 | shootin 1 |
| 8613 | 58.59 | 263.01 | 0.22 | PPAP2B | phosphatidic acid phosphatase type 2B |
| 427 | 223.03 | 1052.38 | 0.21 | ASAH1 | N-acylsphingosine amidohydrolase (acid ceramidase) 1 |
| 6303 | 220.91 | 1245.42 | 0.18 | SAT1 | spermidine/spermine N1-acetyltransferase 1 |
| 55686 | 38.92 | 218.2 | 0.18 | MREG | melanoregulin |
| 125058 | 71.47 | 407.37 | 0.18 | TBC1D16 | TBC1 domain family, member 16 |
| 1846 | 35.73 | 219.22 | 0.16 | DUSP4 | dual specificity phosphatase 4 |
| 4681 | 104.65 | 637.66 | 0.16 | NBL1 | neuroblastoma 1, DAN family BMP antagonist |
| 5627 | 23.42 | 146.83 | 0.16 | PROS1 | protein S (alpha) |
| 2059 | 51.96 | 398.02 | 0.13 | EPS8 | epidermal growth factor receptor pathway substrate 8 |
| 57214 | 29.45 | 224.94 | 0.13 | CEMIP | cell migration inducing protein, hyaluronan binding |
| 2065 | 31.52 | 262.95 | 0.12 | ERBB3 | erb-b2 receptor tyrosine kinase 3 |
| 25805 | 92.13 | 769.91 | 0.12 | BAMBI | BMP and activin membrane-bound inhibitor |
| 79589 | 12.85 | 108.73 | 0.12 | RNF128 | ring finger protein 128, E3 ubiquitin protein ligase |
| 8870 | 115.81 | 1089.83 | 0.11 | IER3 | immediate early response 3 |
| 9289 | 82.06 | 964.38 | 0.085 | ADGRG1 | adhesion G protein-coupled receptor G1 |
| 25797 | 20.41 | 317.31 | 0.064 | QPCT | glutaminyl-peptide cyclotransferase |
| 3958 | 132.2 | 2923.57 | 0.045 | LGALS3 | lectin, galactoside-binding, soluble, 3 |

^1^Mean of intensities in the resistant group

^2^Mean of intensities in the sensitive group

^3^Fold-change of the mean intensities in resistant group / sensitive group
